# Sirtuin 1 activation protects against early brain injury after experimental subarachnoid hemorrhage in rats

**DOI:** 10.1038/cddis.2016.292

**Published:** 2016-10-13

**Authors:** Xiang-Sheng Zhang, Qi Wu, Ling-Yun Wu, Zhen-Nan Ye, Tian-Wei Jiang, Wei Li, Zong Zhuang, Meng-Liang Zhou, Xin Zhang, Chun-Hua Hang

**Affiliations:** 1 Department of Neurosurgery, Jinling Hospital, School of Medicine, Nanjing University, Nanjing, Jiangsu Province, P.R. China; 2 Department of Neurosurgery, Jinling Hospital, School of Medicine, Southern Medical University (Guangzhou), Nanjing, Jiangsu Province, P.R. China

## Abstract

Increasing evidence indicates that sirtuin 1 (SIRT1) is implicated in a wide range of cellular functions, such as oxidative stress, inflammation and apoptosis. The aim of this study was to investigate the change of SIRT1 in the brain after subarachnoid hemorrhage (SAH) and its role on SAH-induced early brain injury (EBI). In the first set of experiments, rats were randomly divided into sham group and SAH groups at 2, 6, 12, 24, 48 and 72 h. The expression of SIRT1 was evaluated by western blot analysis, immunohistochemistry and immunofluorescence. In another set of experiments, SIRT1-specific inhibitor (sirtinol) and activator (activator 3) were exploited to study the role of SIRT1 in SAH-induced EBI. It showed that the protein level of SIRT1 was markedly elevated at the early stage of SAH and peaked at 24 h after SAH. The expression of SIRT1 could be observed in neurons and microglia, and the enhanced SIRT1 was mainly located in neurons after SAH. Administration of sirtinol inhibited the expression and activation of SIRT1 pathways after SAH, while activator 3 enhanced the expression and activation of SIRT1 pathways after SAH. In addition, inhibition of SIRT1 could exacerbate forkhead transcription factors of the O class-, nuclear factor-kappa B- and p53-induced oxidative damage, neuroinflammation and neuronal apoptosis, leading to aggravated brain injury after SAH. In contrast, activator 3 treatment could reduce forkhead transcription factors of the O class-, nuclear factor-kappa B-, and p53-induced oxidative damage, neuroinflammation and neuronal apoptosis to protect against EBI. These results suggest that SIRT1 plays an important role in neuroprotection against EBI after SAH by deacetylation and subsequent inhibition of forkhead transcription factors of the O class-, nuclear factor-kappa B-, and p53-induced oxidative, inflammatory and apoptotic pathways. SIRT1 might be a new promising molecular target for SAH.

Subarachnoid hemorrhage (SAH) is a fatal neurological injury with high morbidity and mortality rates. Accumulating studies indicated that early brain injury (EBI) instead of cerebral vasospasm is acknowledged as the primary cause of a poor outcome for patients with SAH.^1, 2^ Therefore, treatment of EBI has been considered to be the main goal in the management of patients with SAH. However, despite intense researches have been carried out to elucidate its mechanisms, the exact molecular mechanisms of EBI are still not well understood. It has hindered the development of effective and specific treatment paradigms for EBI. Hence, it is a critical need to develop new therapeutic strategies for EBI treatment.

Sirtuins (SIRTs) are a family of deacetylases with homology to *Saccharomyces cerevisiae* silent information regulator 2, which require nicotinamide adenine dinucleotide (NAD^+^) as a cofactor for the deacetylation reaction.^3^ In mammals, seven SIRTs have been identified, and sirtuin 1 (SIRT1) is the best-studied protein from this family.^3^ Multiple lines of studies have revealed that SIRT1 could modulate a variety of biological functions, such as oxidative stress, immune response, mitochondrial biogenesis and apoptosis/autophagy.^4, 5^ In the central nervous system diseases including cerebral ischemia, traumatic brain injury, Alzheimer's disease and Parkinson's disease, SIRT1 has shown protective effects due to its functions in metabolism, stress resistance and genomic stability.^5, 6, 7^ However, until now, the cellular expression and the neuroprotective effects of SIRT1 on SAH-induced EBI remain obscure.

In our previous work, we found that the expression level of SIRT1 was significantly increased after SAH, indicating a possible role of SIRT1 after SAH.^8^ SIRT1 is a member of NAD^+^-dependent protein deacetylases involved in a wide variety of cellular functions by deacetylating its target proteins, including forkhead transcription factors of the O class (FoxOs), nuclear factor-kappa B (NF-кB) and p53.^9, 10^ A growing body of evidence has demonstrated that FoxOs-, NF-кB and p53 play important roles in modulating oxidative, inflammatory and apoptotic processes.^3, 9, 11^ Importantly, a considerable number of experimental studies have demonstrated that SIRT1 can protect neuronal survival against FoxOs-, NF-кB- and p53-induced oxidative, inflammatory and apoptotic pathways.^12, 13, 14, 15^ It seems that elevating SIRT1 activity is a valid target for the treatment of SAH. To address this possibility, we further conducted a study to investigate the changes of SIRT1 expression and its role on SAH-induced EBI, and to explore the possible underlying mechanisms.

## Results

### Mortality rate and general observations

There was no significant difference in arterial blood gas and heart rate among experimental groups. In the first experiment, no animals died in the sham group (0 of 18 rats), and the mortality rate of the rats was 17.2% (10 of 58) after induction of SAH. In the second experiment, the mortality rate at 24 h after surgery was 0% (0 of 18) in the sham group; 18.2% (4 of 22) in the SAH group; 21.7% (5 of 23) in the SAH+vehicle group; 32% (8 of 25) in the SAH+sirtinol group. In the third experiment, the mortality rate at 72 h after surgery was 0% (0 of 12) in the sham group; 20% (3 of 15) in the SAH group; 20% (3 of 15) in the SAH+vehicle group; 33.3% (6 of 18) in the SAH+sirtinol group. In the fourth experiment, the mortality rate at 24 h after surgery was 0% (0 of 18) in the sham group; 21.7% (5 of 23) in the SAH+vehilce group; 16.7% (6 of 36) in the SAH+A3 group.

### Expression of SIRT1 in brain cortex after SAH

**Figure 1:**
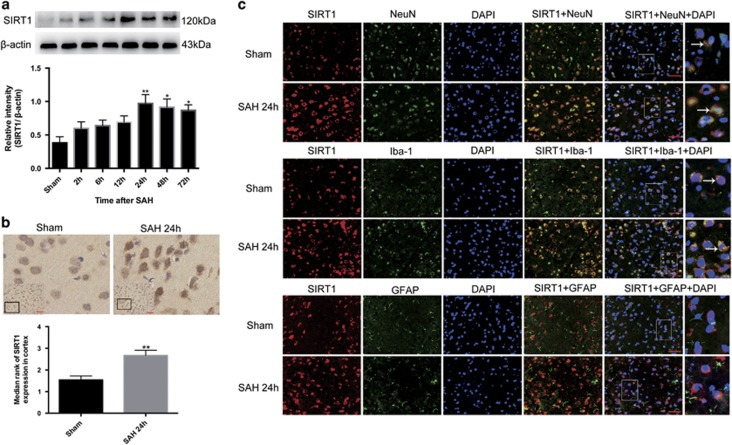
Expression and cellular distribution of SIRT1 in brain cortex after SAH. (**a**) Western blot analysis showed the expression of SIRT1 at 2, 6, 12, 24, 48 and 72 h after SAH.&#8232; (**b**) After experimental SAH, SIRT1 was intensely immunoreactive in both cytoplasm and nuclei. (**c**) Double immunofluorescence staining showed that SIRT1 expressed mostly in neurons but also in microglia but not in astrocytes. Arrows point to SIRT1-positive neurons or SIRT1-positive microglia. *n*=6 in each group. Bars represent the mean±S.E.M. **P<0.01, *P<0.05 versus sham group. Scale bars=50 μm

Western blot was performed to assess the time course of SIRT1 expression after SAH. As shown (Figure 1a), the level of SIRT1 expression was low in the sham group, while it increased significantly and peaked at 24 h and remained ascended at 72 h post SAH. There was a statistically significant difference between the sham group and 24, 48, 72-h groups.

The expression and distribution of SIRT1 was further identified by immunohistochemical staining at the peak activation of SIRT1 (at 24 h after SAH according to western blot). As shown (Figure 1b), SIRT1 was constitutively and weakly expressed in the sham group. In contrast, SIRT1 expression was evidently increased in the cortex at 24 h after SAH. Moreover, both cytosolic and nucleic immunoreactivities of SIRT1 in lots of neural cells were enhanced after SAH.

To identify which kind of brain cells SIRT1 mainly expressed after SAH, double immunofluorescence staining was performed. As shown (Figure 1c) in the sham group, weak expression of SIRT1 in the neurons and microglia were observed. Compared with sham group, SAH enhanced the expression of SIRT1 in both neurons and microglia, especially in neurons. However, SIRT1-positive cells that also were positive for glialfibrillary acidic protein cannot be found in both sham group and SAH group. These results suggested that SIRT1 was expressed in neurons and microglia rather than astrocytes, and the enhanced SIRT1 was mainly located in neurons after SAH.

### Effects of sirtinol treatment on the level of SIRT1 under SAH conditions

**Figure 2:**
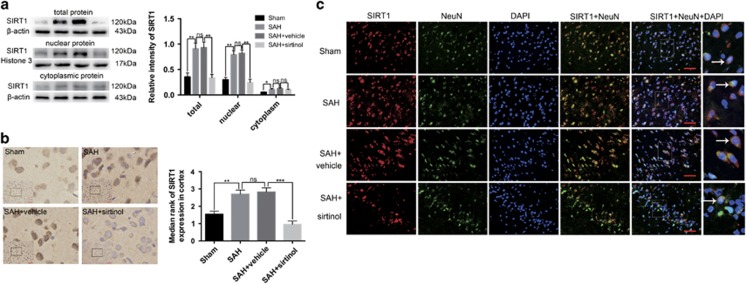
Effects of sirtinol on the expression of SIRT1 at 24 h post SAH. (**a**) Western blot results showed that sirtinol significantly reduced the expression of SIRT1 in both total protein and nuclear protein but had little effects on the cytoplasmic protein of SIRT1. (**b**) SIRT1 immunoreactivity in brain cortex was significantly decreased by sirtinol treatment. (**c**) SAH enhanced the expression of SIRT1 in neurons, which was significantly decreased by sirtinol treatment. Arrows point to SIRT1-positive neurons. *n*=6 in each group. Bars represent the mean±S.E.M. ***P<0.001, **P<0.01, *P<0.05, ^ns^*P*>0.05. Scale bars=50 μm. ns, non-significant

To investigate the potential role of SIRT1 in the EBI, we first evaluated the effects of SIRT1-specific inhibitor sirtinol on the expression of SIRT1 after SAH. As shown (Figure 2a), SAH significantly increased the expression of SIRT1 in total protein, nuclear protein and cytoplasmic protein compared with sham group. In contrast, sirtinol treatment markedly reduced the expression of SIRT1 in both total protein and nuclear protein. However, sirtinol treatment had little effects on the cytoplasmic protein of SIRT1 when compared with SAH+vehicle group. Similarly, immunohistochemical staining (Figure 2b) and double immunofluorescence staining (Figure 2c) revealed elevated SIRT1-positive neurons in the cerebral cortex at 24 h after SAH, which was also decreased by sirtinol treatment.

### Influence of sirtinol on oxidative stress and inflammation at 24 h post SAH

**Figure 3:**
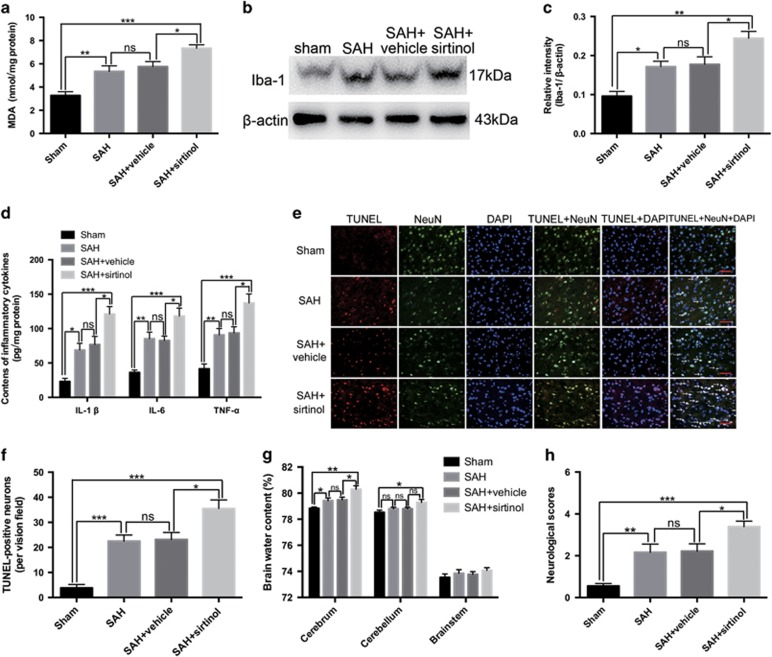
Effects of SIRT1 inhibition on oxidative stress, inflammation, neuronal apoptosis, brain edema and neurological functions at 24 h post SAH. (**a**–**d**) Inhibition of SIRT1 further enhanced the increased levels of MDA, Iba-1, IL-1*β*, IL-6 and TNF-*α* after SAH. (**e**) Representative photomicrographs of TUNEL staining in the experimental groups. Arrows point to TUNEL-positive neurons. (**f**) Sirtinol treatment significantly increased the number of TUNEL-positive neurons after SAH. (**g**) In SAH+sirtinol group, brain water content in the cerebrum was markedly higher than that in the SAH+vehicle group. (**h**) When given sirtinol treatment, the impairment of neurological behaviour was more pronounced. *n*=6 in each group. The values are expressed as mean±S.E.M. ***P<0.001, **P<0.01, *P<0.05, ^ns^*P*>0.05. Scale bars=50 μm

Malondialdehyde (MDA), the end product of lipid peroxidation of polyunsaturated fatty acids in cellular membranes, was identified as a reliable marker of oxidative stress.^16^ As shown (Figure 3a), the content of MDA increased significantly after SAH as compared with the sham group, which was markedly further elevated after treatment with sirtinol.

Activation of microglia is a critical source of pro-inflammatory cytokines in the brain. As shown (Figures 3b–d), SAH significantly increased the expression of Iba-1, and production of IL-1*β*, IL-6 and TNF-*α* when compared with sham group. Inhibition of SIRT1 further enhanced the increased levels of Iba-1, IL-1*β*, IL-6 and TNF-*α* after SAH.

### Effects of sirtinol on neuronal apoptosis, brain edema and neurological function at 24 h post SAH

SIRT1 regulates apoptosis under pathophysiological conditions.^17^ We evaluated the effects of sirtinol on neuronal apoptosis by TUNEL staining. As shown (Figures 3e and f), only a few TUNEL-positive neurons in the basal temporal lobe were observed in the sham group. More TUNEL-positive neurons appeared in the SAH and SAH+vehicle groups. After sirtinol treatment, the percentage of TUNEL-positive neurons was further enhanced as compared with the SAH+vehicle group.

Brain edema is an independent risk factor for poor outcome and death after SAH. As shown (Figure 3g), the water contents in different brain regions of sham group were relatively low. A significant increase in water content in the cerebrum was observed in the SAH and SAH+vehicle groups when compared with that in the sham group. Suppression of SIRT1 by sirtinol further increased the water content in the cerebrum as compared with SAH+vehicle group. In addition, SIRT1 inhibition significantly increased the water content in the cerebellum as compared with that in sham group. We next investigated whether SIRT1 inhibition could affect neurological outcomes after SAH. Consistently, SAH insults caused a worse neurological function compared with sham group at 24 h after surgery. After SIRT1 inhibition by sirtinol, the impairment of neurological function was more pronounced than that in the SAH+vehicle group (Figure 3h).

### Effects of sirtinol on SIRT1 signalling pathways after SAH

**Figure 4:**
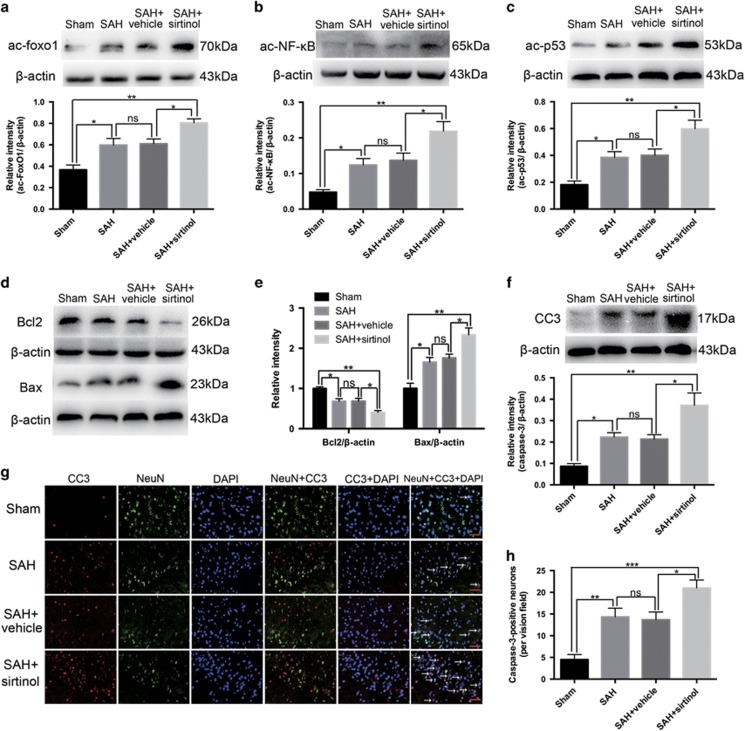
Effects of sirtinol on SIRT1 signalling pathways after SAH. (**a**–**c**) Sirtinol significantly increased ac-FoxO1, ac-NF-кB and ac-p53 expression after SAH. (**d**–**f**) Inhibition of SIRT1 enhanced apoptotic pathway, including significantly increased the levels of cleaved caspase-3 and Bax, and diminished the expression of Bcl2. (**g**) Representative photomicrographs of caspase-3 staining in the experimental groups. Arrows point to caspase-3 positive neurons. (**h**) Suppression of SIRT1 increased the number of caspase-3 positive neurons. *n*=6 in each group. Bars represent the mean±S.E.M. ***P <0.001, **P<0.01, *P<0.05, ^ns^*P*>0.05. Scale bars=50 μm

SIRT1 is a member of NAD; dependent protein deacetylases, with a vast list of substrate proteins including the FoxOs, NF-кB and p53.^12^ To investigate whether sirtinol acts through regulating SIRT1 pathways, we evaluated the levels of ac-FoxO1, ac-NF-кB and ac-p53 by western blot. As shown (Figures 4a–c), SAH insults could significantly increase the levels of ac-FoxO1, ac-NF-кB and ac-p53 when compared with the sham group. After administration with sirtinol, the increased ac-FoxO1, ac-NF-кB and ac-p53 expression was markedly further elevated as compared with SAH+vehicle group. To better understand the underlying mechanisms of neuronal deterioration after inhibition the SIRT1, the apoptotic pathways were evaluated. As shown (Figures 4d–f), compared with the vehicle-treated group, suppression of SIRT1 by sirtinol significantly enhanced apoptosis with elevated levels of cleaved caspase-3 and pro-apoptotic protein Bax, and diminished the level of Bcl2. In addition, SIRT1 inhibition significantly increased the number of caspase-3-positive neurons as compared with SAH+vehicle group (Figures 4g and h). Taken together, these data suggested that inhibition of SIRT1 could amplify oxidative, inflammatory and apoptotic pathways following SAH.

### Effects of sirtinol on neuronal survival, brain water content and neurological function at 72 h post SAH

**Figure 5:**
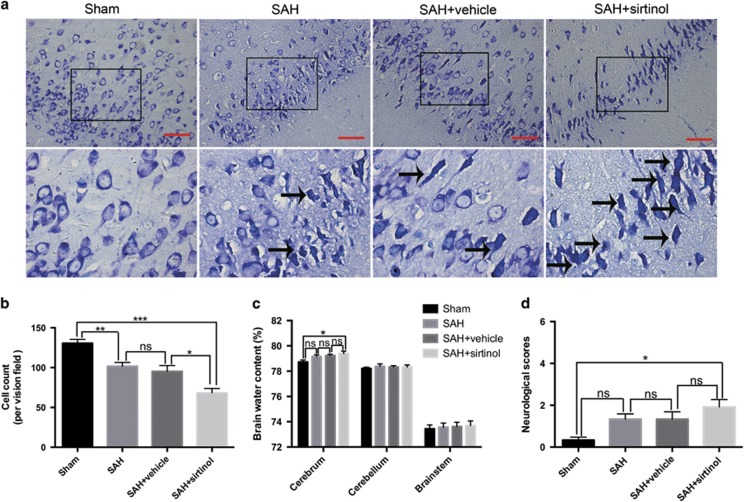
Effects of sirtinol on neuronal survival, brain edema and neurological function at 72 h post SAH. (**a**) Representative photomicrographs of Nissl staining in the experimental groups. Arrows point to dead neurons. (**b**) Suppression of SIRT1 by sirtinol significantly reduced the number of survived neurons. (**c**) The brain water content was markedly higher in the SAH+sirtinol group than that in the sham group. However, there was no significant difference between SAH+vehicle and SAH+sirtinol groups. (**d**) SIRT1 inhibition caused a worse neurological function compared with sham group. Bars represent the mean±S.E.M. ***P<0.001, **P<0.01, *P<0.05, ^ns^*P*>0.05. Scale bars=50 μm

For a better understanding of the comprehensive effects of SIRT1 inhibition on EBI following SAH, neuronal survival, brain water content and neurological function were further evaluated at 72 h post SAH. As shown (Figures 5a and b), SIRT1 inhibition significantly decreased the proportion of surviving neurons. In parallel, the brain water content in the cerebrum was markedly increased after SIRT1 inhibition when compared with sham group (Figure 5c). In the mean time, sirtinol treatment rats displayed a worse neurological function as compared with sham group. There was no significant difference in the neurological functions among sham, SAH and SAH+vehicle groups at 72 h post SAH (Figure 5d).

### Effects of A3 on the expression and activation of SIRT1 pathways after SAH

**Figure 6:**
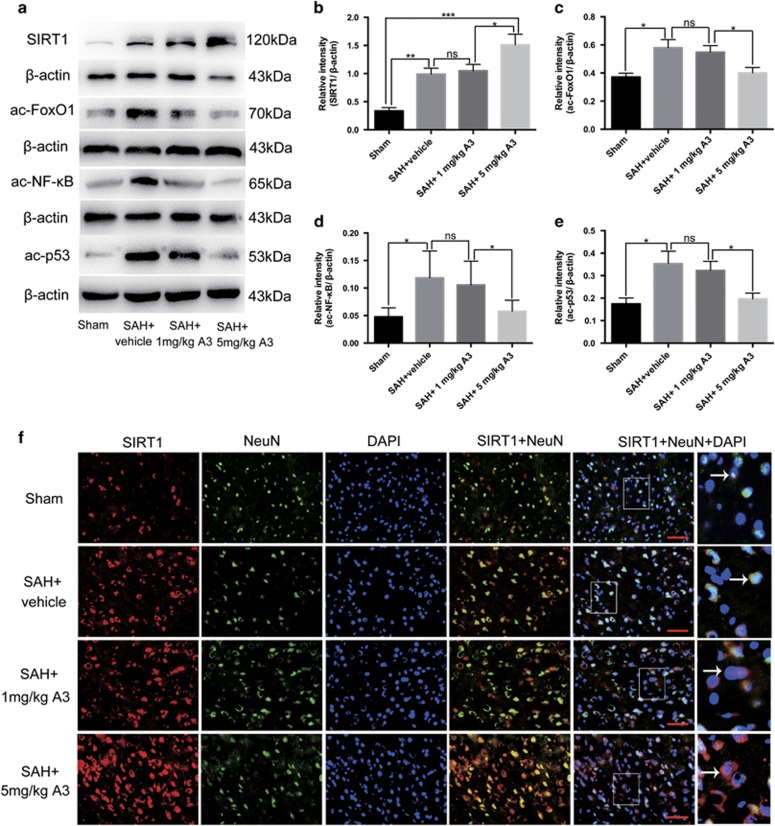
Effects of A3 on the expression and activation of SIRT1 pathways 24 h post SAH. (**a**) Representative images of western blots to detect the effects of SIRT1 activator A3 on the expression of SIRT1, ac-FoxO1, ac-NF-кB and ac-p53. (**b**–**e**) The levels of SIRT1, ac-FoxO1, ac-NF-кBand ac-p53 were significantly increased after SAH, which was evidently decreased by high dose A3 treatment. (**f**) Immunofluorescence labelling showing that high dose A3 treatment significantly increased SIRT1 expression in neurons compared with the SAH+vehicle group. Arrows point to SIRT1-positive neurons. Bars represent the mean±S.E.M. ***P<0.001, **P<0.01, *P<0.05, ^ns^*P*>0.05. Scale bars=50 μm

As shown (Figures 6a, f), both western blot and immunofluorescent staining showed that high dose A3 treatment significantly increased the protein level of SIRT1 compared with SAH+vehicle group. In addition, administration of high dose A3 significantly activated SIRT1 pathways by decreasing the expression of downstream substrates ac-FoxO1, ac-NF-кB and ac-p53. However, A3 at 1 mg/kg had no significant effect (Figures 6c–e). Therefore, we selected 5 mg/kg for further experiments.

### Effects of A3 on oxidative stress, inflammation, neuronal apoptosis, brain water content and neurological function after SAH

**Figure 7:**
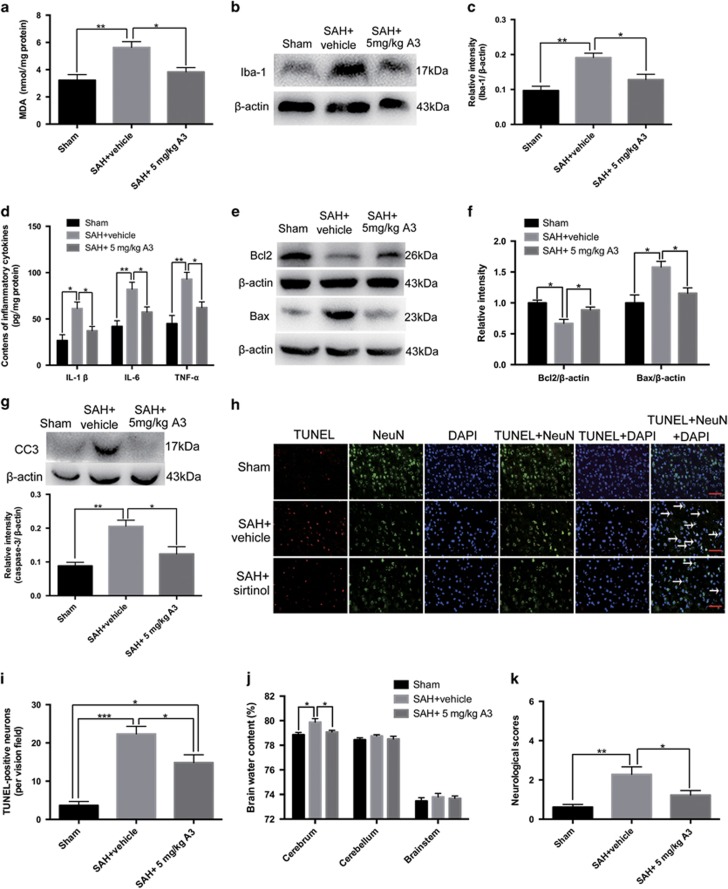
Effects of A3 on apoptosis, oxidative stress, inflammation, brain edema and neurological function at 24 h post SAH. (**a–d**) SIRT1 activation by A3 treatment markedly reduced the increased levels of MDA, Iba-1, IL-1*β*, IL-6 and TNF-*α* at 24 h post SAH. (**e–g**) A3 treatment significantly decreased the protein levels of Bax and caspase-3, and increased the protein level of Bcl2 compared with SAH+vehicle group. (**h**) Representative photomicrographs of TUNEL staining in the experimental groups. Arrows point to TUNEL-positive neurons. (**i**) Activation of SIRT1 by A3 significantly reduced neuronal apoptosis at 24 h post SAH. (**j**, **k**) After A3 administration, the brain edema and neurological behaviour impairment were significantly ameliorated compared with SAH+vehicle group. Bars represent the mean±S.E.M. ***P<0.001, **P<0.01, *P<0.05. Scale bars=50 μm

Consistent with the results above, the content of MDA, Iba-1 expression, and the levels of IL-1*β*, IL-6, and TNF-*α* significantly increased after SAH (Figures 7a–d). A3 treatment markedly reduced the content of MDA, Iba-1 expression, and the levels of IL-1*β*, IL-6, and TNF-*α* as compared with SAH+vehicle group (Figures 7a–d). The expressions of apoptotic proteins were assessed by western blot. It showed that A3 treatment could significantly decrease the levels of Bax and cleaved caspase-3, and increase the expression of Bcl2 (Figures 7e–g). In addition, TUNEL-positive neurons were also reduced after treatment of A3 at 24 h after SAH (Figures 7h and i). We next investigated the effects of A3 on brain edema and neurological function after SAH. Results showed that treatment with A3 could dramatically ameliorate the impairment of neurologic behaviour and brain edema in the cerebellum as compared with SAH+vehicle group (Figures 7j and k). These results suggested that activation of SIRT1 might protect against EBI.

## Discussion

**Figure 8:**
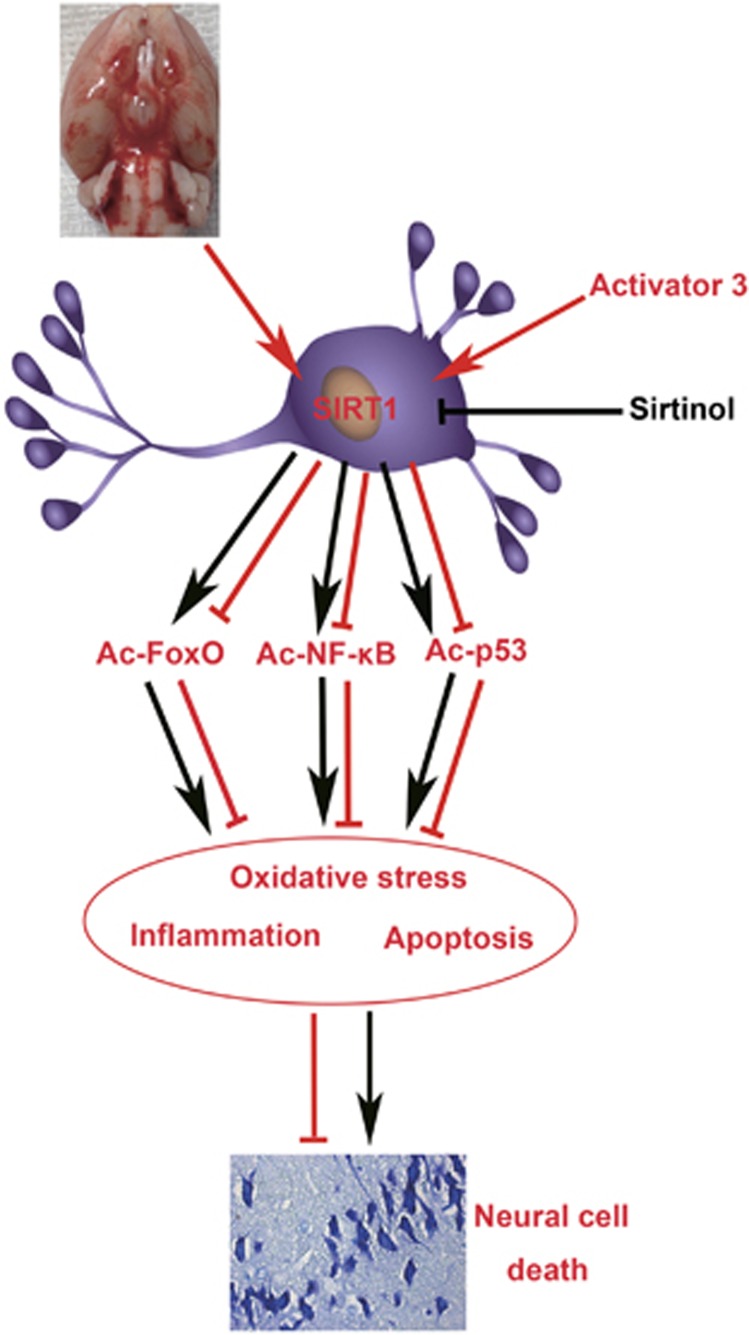
Schematic illustrating the possible mechanisms of neuroprotective role of SIRT1 in EBI after SAH. As illustrated, SAH insults cause an increase in SIRT1 expression in neurons, leading to ac-FoxO, ac-NF-кB and ac-p53 deacetylation, which prevent the EBI. Inhibition of SIRT1 by sirtinol will block the signal transduction to induce neural cell death, eventually aggravate EBI following SAH. In contrast, activation SITR1 by activator 3 will stimulate the signal transduction to prevent EBI. The red and black arrows indicate direct or indirect induction, while the red and black blocked lines indicate direct or indirect repression

In the present study, we demonstrated that SIRT1 was upregulated in the brain cortex after SAH. The levels of SIRT1 protein increased in a time-dependent manner, and peaked at 24 h after SAH. Immunohistochemistry and immunofluorescence studies showed that SIRT1 expressed mostly in neurons as well as in microglia but not in astrocytes, and that SAH enhanced the expression of SIRT1 in neurons. Additionally, SIRT1 inhibition by sirtinol could exacerbate brain injury by increasing acetylation and subsequent activation of FoxOs-, NF-кB- and p53-induced oxidative stress, neuroinflammation and apoptosis. In contrast, activation of SIRT1 by A3 could ameliorate EBI concomitant with decreased acetylation of FoxOs, NF-кB and p53. It suggested that SIRT1 might play an endogenous brain protection role after SAH by deacetylation and subsequent inhibition FoxOs-, NF-кB- and p53-induced oxidative, inflammatory and apoptotic pathways (Figure 8).

SIRT1 is a type of histone deacetylase whose activity is dependent on NAD^+^. SIRT1 has been reported as an important regulator to modulate transcription, apoptosis, cell survival, DNA repair, inflammation and oxidative stress through the deacetylation of intracellular signalling molecules and chromatin histones.^18^ In recent years, considerable attention has been attached to SIRT1 in central nervous system diseases since it is considered as a promising target in a variety of acute and chronic neurodegenerative diseases.^10, 13, 19^ It has been demonstrated that SIRT1 overexpression or activation is protective in neurodegenerative diseases and acute nervous system injury, including brain ischemia and traumatic brain injury.^12, 13, 14^ In the brain, it has been proved that SIRT1 is expressed with high levels in the cortex, hippocampus, cerebellum and hypothalamus.^4^ Meanwhile, SIRT1 has been implicated in neuronal plasticity, neuronal death and cognitive functions.^4, 10, 20^ However, to date, few studies are conducted to elucidate the potential role of SIRT1 in the SAH.

In the experiment of our study, we first evaluated the time course of SIRT1 expression in the early period after SAH. Our results suggested that SAH insults could increase SIRT1 protein expression in the cerebral cortex with a peak at 24 h after SAH. Meanwhile, the immunohistochemistry study also revealed a strong correspondence with the degree of SIRT1 expression in the experimental groups. At the cellular level, SIRT1 is known as a nuclear protein, which is predominantly expressed in neurons.^21^ In the current study, we further performed double immunofluorescent staining to evaluate the cellular distribution of SIRT1. Consistent with previous studies,^4, 12, 19^ we showed that SIRT1 could be expressed in neurons and microglia, and the elevated SIRT1 after SAH was mainly located in the neurons in the rat brain cortex. Moreover, both cytosolic and nucleic immunoreactivities of SIRT1 in neurons and microglia were enhanced after SAH. These evidences indicated that elevated SIRT1 in activated neural cells might play an important role in the EBI. However, it should be noted that we could not find glialfibrillary acidic protein-positive astrocytes that also be stained with SIRT1 antibody either in the sham group or in the SAH group. This result was in agreement with Hernandez-Jimenez *et al*'s work,^12^ in which they found that no SIRT1 was located in astrocytes in a permanent middle cerebral artery occlusion mice model. While, recent studies have reported that SIRT1 can be expressed in cultured rat or mice astrocytes.^22, 23^ The reason for the discrepancy is unknown at this time. One possible explanation could be that anti-SIRT1 antibody from different species might affect the subcellular localization of SIRT1 in different animal species. However, additional experiments are still needed to unravel this issue.

It has been demonstrated that inflammatory response and oxidative stress are closely associated with EBI following SAH.^1, 2^ In the current study, we further evaluated the potential roles of SIRT1 in the EBI with its specific inhibitor and activator. We observed that SIRT1 inhibitor sirtinol could significantly reduce the expression of SIRT1 in both total protein and nuclear protein. However, sirtinol treatment had little effects on the cytoplasmic protein of SIRT1. These findings suggested that the influence of sirtinol on SIRT1 was mainly at its nuclear level. Concomitant with the decreased levels of SIRT1, the microglia activation and pro-inflammatory cytokines release, and aggravated oxidative damage in the cortex were further amplified. These results were in accordance with previous studies demonstrating that SIRT1 could protect cells against various stresses, including inflammation and oxidative stress.^3, 12, 24^ In addition, our results showed that inhibition SIRT1 could exacerbate neuronal apoptosis, brain edema and neurological deterioration at 24 h after SAH. To confirm whether activation of SIRT1 also confers brain protection, we further used SIRT1 activator in another set of experiments. As expected, we demonstrated that high dose A3 could efficiently reduce neuroinflammation and oxidative damage, and ameliorate neuronal apoptosis, brain edema and neurological deterioration after SAH. Altogether, these results strongly support the neuroprotective role of SIRT1 in the EBI following SAH. However, we noted that at 72 h post SAH, inhibition of SIRT1 neither enhanced brain water content nor aggravated neurological deficits. It is unclear at this time whether the dose and duration of the sirtinol treatment are not sufficient to discriminate the statistical difference in different groups. Multiple and prolonged use of sirtinol treatment may be warranted for the future study of SIRT1.

Regarding the mechanisms involved in SIRT1-induced neuroprotection, numerous deacetylation substrates are participated in.^19^ Acetylation is a post-translational modification that regulates protein function. Not only histones but also transcription factors and cytoplasmic proteins undergo lysine acetylation.^19^ SIRT1 can deacetylate a variety of substrates, including FoxOs, NF-кB, p53, endothelia nitric oxide synthase, peroxisome proliferator-activated receptor-gamma (PPAR*γ*), and PPAR*γ* coactivator-1*α* that are all potentially important for neuronal survival.^10, 13^ For example, SIRT1 ameliorates oxidative stress through the modulation of nuclear shuttling and transcriptional activity of FoxOs. Dependent upon the post-translational changes on FoxOs, SIRT1 can control FoxOs activity to protect cells from oxidative damage or lead to gene activation.^9^ In addition, SIRT1 can directly interact with RelA/p65 subunit of NF-кB, and deacetylate ly310 residue of RelA/p65 to suppress NF-кB transcriptional activity leading to down-regulation of pro-inflammatory cytokines gene expression.^3^ Moreover, SIRT1 can deacetylate p53 and prevent p53-mediated transcriptional activity to block apoptosis.^9, 15^ In agreement with these findings above, our results demonstrated that inhibition of SIRT1 resulted in enhanced acetylation of FoxO1, NF-кB and p53 in the brain. Concomitant with the increased acetylation of FoxO1, NF-кB and p53, the oxidative, inflammatory and apoptotic pathways were activated after SIRT1 inhibition. In contrast, treatment with A3 significantly increased deacetylation of these proteins leading to neuroprotective actions in EBI. Given that these proteins play critical roles in SAH physiology,^25, 26, 27^ our results suggested that SIRT1 conferred neuroprotection against EBI via deacetylation and subsequent inhibition of FoxOs-, NF-кB- and p53-induced oxidative, inflammatory and apoptotic pathways. However, it should be noted that the increased level of SIRT1 had not significantly reduced the deacetylation levels of FoxO1, NF-кB and p53 in the SAH group. In contrast, the protein levels of ac-FoxO1, ac-NF-кB and ac-p53 were elevated in the SAH group. It may suggest that in addition to SIRT1, other deacetylases may be involved in the pathogenesis of EBI. Therefore, future work remains to be conducted to unravel this novel area.

At the time of study, we noted some other discrepancies about the potential roles of SIRT1 in different fields. There is a wealth of studies that have shown that the expression of SIRT1 in the brain is increased in the models of cerebral ischemia and traumatic brain injury.^12, 14, 28^ However, some other studies demonstrated that the protein level of SIRT1 is decreased after cerebral ischemia, myocardial ischemia/reperfusion or septic brain injury.^29, 30, 31^ In addition, most of the previous studies have concluded that activation or overexpression SIRT1 is advantageous in various diseases.^17, 19^ But the detrimental effects of SIRT1 have also been reported.^32, 33^ For example, Li *et al* demonstrated that SIRT1 inhibition could prevent cell death in cultured neurons subjected to oxidative stress.^34^ Jin *et al* found that cytoplasm-localized SIRT1 could enhance apoptosis sensitivity although the exact mechanisms remain obscure.^35^ The reasons for these disagreements are unclear at this time. One possible explanation could be that the pathophysiology of SAH is different from other diseases. Different subcellular localization, animal species, degree of damage or administration routes might affect actions of SIRT1 in different diseases.

There are several limitations in our study. First, SIRT1 activation has numerous biological functions.^10, 17^ We cannot exclude the possibility that other functions of SIRT1 activation might play roles in the development of EBI. In addition, the molecular basis underlying SIRT1 upregulation induced by SAH is still obscure. It has been demonstrated that several stimuli can regulate the expression of SIRT1 at the transcriptional levels, and notably, one of the main triggers is oxidative stress.^17^ In response to changes in environment, E2F transcription factor 1, tumour suppressor hypermethylated in cancer 1 and FoxO have been identified to modulate SIRT1 transcription.^17^ However, whether they are attributable to the increased expression of SIRT1 after SAH remain obscure. Given the current research is a pilot study, further studies are still needed to validate the exact role of SIRT1 following SAH.

In conclusion, our study demonstrates that SIRT1 plays an important role in endogenous neuroprotection by deacetylation and subsequent inhibition of FoxOs-, NF-кB- and p53-induced oxidative, inflammatory and apoptotic pathways. Although more work is required, current data shed new light on the treatment of SAH, and suggest that activation SIRT1 may be an effective therapeutic strategy for EBI treatment.

## Materials and Methods

### Animal preparation

All procedures were approved by the Animal Care and Use Committee of Nanjing University and were conformed to Guide for the Care and Use of Laboratory Animals by National Institutes of Health. Adult male Sprague-Dawley rats (250–300 g) were purchased from Animal Center of Jinling Hospital. They were acclimated in a reversed 12-h light/12-h dark cycle controlled environment with free access to food and water.

### Experimental SAH model

Experimental SAH model was produced according to previous studies.^36, 37^ Briefly, after intraperitoneal anesthesia with 10% chloral hydrate (0.35 ml/100 g), the rats were positioned prone in a stereotactic frame. The amount of 0.35 ml of non-heparinized fresh autologous arterial blood from the femoral artery was slowly (in the course of 20 s) injected into the prechiasmatic cistern with a syringe pump under aseptic conditions. After injection, animals were kept in a 30 °C, heads-down position for 20 min. Animals in the sham group were injected with 0.35 ml saline. Then, 2 ml saline was injected subcutaneously right after the operation. After the procedures, the rats were returned to their cages individually, and food and water were kept easily accessible. Heart rate and rectal temperature were monitored, and the rectal temperature was kept at 37 °C±0.5 °C by using a warm pad when required, throughout the experiments.

### Experiment design

A schematic of experimental protocols is given in Supplementary Figure S1.

Experiment design 1 – A total of 76 rats were used in this experiment. Of them, 10 animals died after operation. Those animals were excluded from further analysis. Thirty-six rats with SAH were randomly divided into six subgroups and killed by ventricle perfusion at 2, 6, 12, 24, 48 and 72 h post SAH (*n*=6 each). Another 12 rats with SAH were selected randomly for immunohistochemistry and immunofluorescence study at the peak activation of SIRT1 (*n*=6 for each study). Eighteen sham animals underwent the same surgery process with the exception that no blood was injected into the prechiasmatic cistern. In our previous studies, we found that there was no statistical difference of all detected variables among sham groups at each time point.^38, 39^ Therefore, animals in sham group were sacrificed at 24 h after sham operation. Schematic representation of the areas taken for assay was shown in Supplementary Figure S2.

Experiment design 2 – To determine the role of SIRT1 in the EBI after SAH, 72 rats (89 rats were used, 17 rats died) were randomly divided into four groups: sham, SAH, SAH+vehicle and SAH+sirtinol (*n*=18 each). All the rats were killed at 24 h after SAH according to the results of the first experiment. Post assessments included neurological function, brain water content, western blot, biochemical analysis, ELISA and histopathological study.

Experiment design 3 – A separate cohort of rats was used to evaluate the effects of inhibition of SIRT1 on EBI at 72 h post SAH. Forty-eight rats (60 rats were used, 12 rats died) were randomly assigned into four groups: sham, SAH, SAH+vehicle and SAH+sirtinol (*n*=12 each). Post assessments included Nissl staining, brain water content and neurological function.

Experiment design 4 – To confirm whether activation of SIRT1 confers brain protection, SIRT1 activator was exploited. Sixty-six rats (77 rats were used, 11 rats died) were randomly assigned into the following groups: sham group, SAH+vehicle group and SAH+activator 3 (A3) group. All the rats were killed at 24 h post SAH. Post assessments included neurological function, brain water content, western blot, biochemical analysis, ELISA and histopathological study.

### Drug administration

Sirtinol (Sigma, St. Louis, MO, USA) was diluted in vehicle (dimethylsulfoxide, DMSO) (Sigma) at concentration of 2 mmol/l. Sirtinol (2 mmol/l, 30 μl/kg) or vehicle (30 μl/kg) was administrated into the left lateral ventricle (0.8 mm posterior and 1.5 mm lateral to the bregma, and 3.7 mm below the dural layer) through a 25 μl Hamilton syringe (Shanghai Gaoge Industry & Trade Co., Ltd., Shanghai, China) 2 h before SAH was induced. The dose of sirtinol was selected according to our previous study.^8^

A3 (Santa Cruz Bio-Technology, Santa Cruz, CA, USA) was diluted at 1 mg/kg and 5 mg/kg in 10% DMSO solution. Rats were treated intraperitoneally with A3 at 30 min and 12 h after SAH induction. The A3 doses were determined based on a previous work.^12^

### Total/cytosolic/nuclear protein extraction

After euthanasia of the rats, the same part of the inferior basal temporal lobe was isolated and processed as described previously.^36^ To extract total protein, brain samples (about 100 mg) were mechanically lysed in 20 mmol/l Tris (pH 7.6) (Sigma). Homogenates were centrifuged at 14 000 g at 4 °C for 15 min. The supernatant was collected and stored at −80 °C for western blot analysis. To extract cytosolic protein, proper brain samples (about 100 mg) were mechanically lysed in 1 ml ice-cold buffer A, which consisted of 10 mmol/l HEPES (pH 7.9), 2 mmol/l MgCl_2_, 10 mmol/l KCl, 0.1 mmol/l EDTA, 1 mmol/l dithiothreitol and 0.5 mmol/l phenylmethylsulphonyl fluoride (all from Sigma). Then 30 μl of 10% NonidetP-40 solution (Sigma) was added to the system. After the mixture was vortexed for 30 s and spun by centrifugation for 10 min at 5000 g, 4 °C, the cytosolic fraction extracts were collected and stored at −80 °C for western blot analysis. The crude nuclear pellets were suspended in 200 μl ice-cold buffer B, which contained 20 mmol/l HEPES (pH 7.9), 1.5 mmol/l MgCl_2_, 420 mmol/l NaCl, 0.1 mmol/l EDTA, 1 mmol/l dithiothreitol, 0.5 mmol/l phenylmethylsulphonyl fluoride and 25% (v/v) glycerol. The mixture was centrifuged at 14 000 g at 4 °C for 15 min. The supernatant containing nuclear proteins was collected and stored at −80 °C for western blot analysis.^40^ Protein concentrations were determined using the BCA method (Beyotime Biotec, Jiangsu, China).

### Western blot analysis

For western blot analysis, equal protein amounts per lane were separated by 10% SDS-polyacrylamide gels and transferred to a polyvinylidenedifluoride membrane. The membrane was blocked in 5% skim milk for 2 h at room temperature and incubated overnight at 4 °C with primary antibodies against SIRT1 (cat# sc-15404, 1:200, Santa Cruz, CA, USA), Iba-1 (cat# sc-98468, 1:200, Santa Cruz, CA, USA), ac-FoxO1 (cat# sc-49437, 1:200, Santa Cruz, CA, USA), ac-p53 (cat# 2570, 1:400, Cell Signaling, MA, USA), ac-NF-кB (cat# 12629 S, 1:400, Cell Signaling), Bcl2 (cat# sc-492, 1:200, Santa Cruz, CA, USA), Bax (cat# sc-493, 1:200, Santa Cruz, CA, USA), caspase-3 (cat# 9661, 1:400, Cell Signaling), histone 3 (cat# BS7416, 1:2000, Bioworld, MN, USA) and *β*-actin (cat# AP0060, 1:5000, Bioworld) in tris-buffered saline with Tween-20 containing 5% skim milk. After the membrane was washed, it was incubated with horseradish peroxidase-conjugated IgG for 2 h at room temperature. The blotted protein bands were visualized by enhanced chemiluminescence western blot detection reagents (Thermo Scientific, Kalamazoo, MI, USA) and were exposed to X-ray film. The quantification of band density was performed using the UN-Scan-It 6.1 software (Silk Scientific Inc., Orem, UT, USA).

### Immunohistochemical staining

For immunohistochemistry, brain sections were incubated overnight at 4 °C with primary antibody against SIRT1 (cat# sc-15404, 1:50, Santa Cruz, CA, USA) followed by a 15-min wash in phosphate buffered saline (PBS, pH 7.4). After that the sections were incubated with horseradish peroxidase conjugated IgG for 60 min at room temperature. After washing for half an hour, diaminobenzidine was used as a chromogen and counterstaining was done with hematoxylin. The negative control was performed without adding SIRT1 antibody, and the other steps were the same between the experiment sections and negative control. Microscopy of the immunohistochemically stained tissue sections was conducted by two pathologists blinded to the grouping. Evaluation of sections was undertaken by assessing the intensity of staining (five grades). ‘0' indicates that there were no detectable positive cells; ‘1' indicates very low density of positive cells; ‘2' indicates a moderate density of positive cells; ‘3' indicates the higher, but not maximal density of positive cells; and ‘4' indicates the highest density of positive cells.

### Immunofluorescence staining

Immunofluorescence staining was performed following the procedures of our laboratory.^39^ Briefly, brain tissue was sliced (6 μm) and blocked with 5% normal fetal bovine serum in PBS containing 0.1% Triton X-100 for 1 h at room temperature prior to incubation with primary antibody overnight at 4 °C. After sections were washed three times with PBS for 45 min, they were incubated with proper secondary antibodies (Alexa Fluor 488 and Alexa Fluor 594, 1:200) for 2 h at room temperature. The slides were washed with PBS again three times for 45 min prior to be counterstained by 4,6-diamidino-2-phenylindole (DAPI) for 2 min. After three washes again, the slides were covered by microscopic glass with anti-fade mounting medium for further study. Negative controls were prepared by omitting the primary antibodies. Fluorescence microscopy imaging was performed using ZEISS HB050 inverted microscope system and handled by Image-Pro Plus 6.0 software (Media Cybernetics, Rockville, MD, USA) and Adobe Photoshop CS5 (Adobe Systems, San Jose, CA, USA).

### Biochemical estimation

Malondialdehyde (MDA) levels were determined according to the manufacturer's instructions (Nanjing Jiancheng Bioengineering Institute, Nanjing, China). The principle of the assay depends on the reaction of lipid peroxidation products with thiobarbituric acid and formation of products named as thiobarbituric acid reacting substances, which give maximum absorbance at 535 nm wavelength. MDA concentrations were given as nmol/mg protein.

### Enzyme-linked immuosorbent assay (ELISA)

The levels of IL-1*β*, IL-6 and TNF-*α* in brain tissue were quantified using specific ELISA kits for rats (Biocalvin Company, Suzhou, China) according to the manufacturers' instructions. The protein concentrations were determined using the BCA method (Beyotime Biotec, Jiangsu, China). Values are expressed as picogram per milligram protein.

### TUNEL staining

Terminal deoxynucleotidyl transferase-mediated dUTP nick-end labelling (TUNEL) staining&#8232;was conducted by using a TUNEL detection kit according to the manufacturer's instructions (Roche Inc., Indianapolis, USA). Briefly, each section was incubated with primary antibody against NeuN (1:100, Millipore, MA, USA) at 4 °C overnight. After sections were washed three times with PBS for 45 min, they were incubated with TUNEL reaction mixture for 1 h at 37 °C in the dark. The slides were washed with PBS again three times for 45 min prior to be counterstained by DAPI for 2 min. After three washes again, the slides were covered by microscopic glass with anti-fade mounting medium for further study. Fluorescence microscopy imaging was performed using ZEISS HB050 inverted microscope system. The positive cells were identified, counted and analysed by two investigators blinded to the grouping. The extent of brain damage was evaluated by the apoptotic index, defined as the average percentage of TUNEL-positive cells in each section counted in 10 cortical microscopic fields (at × 400 magnification). A total of four sections from each animal were used for quantification. The final average percentage of TUNEL-positive cells of the four sections was regarded as the data for each sample.

### Nissl staining

Tissue sections were stained with cresyl violet as previously described.^36^ The sections were hydrated in 1% toluidine blue for 10 min. After washing with double-distilled water, they were dehydrated and mounted with permount. Normal neurons have a relatively big cell body, rich in cytoplasm, with one or two big round nuclei. In contrast, damaged cells show shrunken cell bodies, condensed nuclei, dark cytoplasm and many empty vesicles. To quantify the amount of Nissl staining, 10 random high-power fields (at × 400 magnification) in each coronary section were chosen, and the mean number of intact neurons in the 10 views was regarded as the data of each section. A total of four sections from each animal were used for quantification. The final average number of the four sections was regarded as the data for each sample.

### Brain water content

Brain water content was measured at 24 and 72 h after surgery. Rats were anesthetized and decapitated, and the brains were separated into cerebrum, cerebellum and brain stem. Each part was weighed immediately after removal (wet weight) and after drying in 80 °C for 72 h (dry weight) and the percentage of water content was calculated as [(wet weight − dry weight)/wet weight] × 100%.

### Clinical evaluation

Clinical scores were recorded 24 and 72 h before euthanization based on the independent observations by two independent researchers who were blind to the experimental groups. Three behavioural activity examinations (Table 1) including appetite, activity and neurological deficits were used in the scoring methodology.^36^

### Statistical analysis

All data were presented as mean±S.E.M. The measurements were subjected to one-way analysis of variance followed by Tukey's *post hoc* test. In the semiquantitative analysis of immunohistochemical staining, differences between two experimental groups were determined by the Student's *t*-test. Statistical significance was inferred at *P*<0.05.

## Figures and Tables

**Table 1:** Behaviour scores

**Category**	**Behaviour**	**Score**
Appetite	Finished meal	0
	Left meal unfinished	1
	Scarcely ate	2
		
Activity	Walk and reach at least three corners of the cage	0
	Walk with some stimulation	1
	Almost always lying down	2
		
Deficits	No deficits	0
	Unstable walk	1
	Impossible to walk	2

## Abbreviations

SIRT1: sirtuin 1
EBI: early brain injury
SAH: subarachnoid hemorrhage
SIRTs: sirtuins
NAD^+^: nicotinamide adenine dinucleotide
FoxOs: forkhead transcription factors of the O class
NF-кB: nuclear factor-kappa B
DMSO: dimethylsulfoxide
A3: activator 3
DAPI: 4,6-diamidino-2-phenylindole
PBS: phosphate buffered saline
MDA: malondialdehyde
ELISA: enzyme-linked immuosorbent assay
TUNEL: terminal deoxynucleotidyl transferase-mediated dUTP nick-end labeling
PPAR*γ*: peroxisome proliferator-activated receptor-gamma
