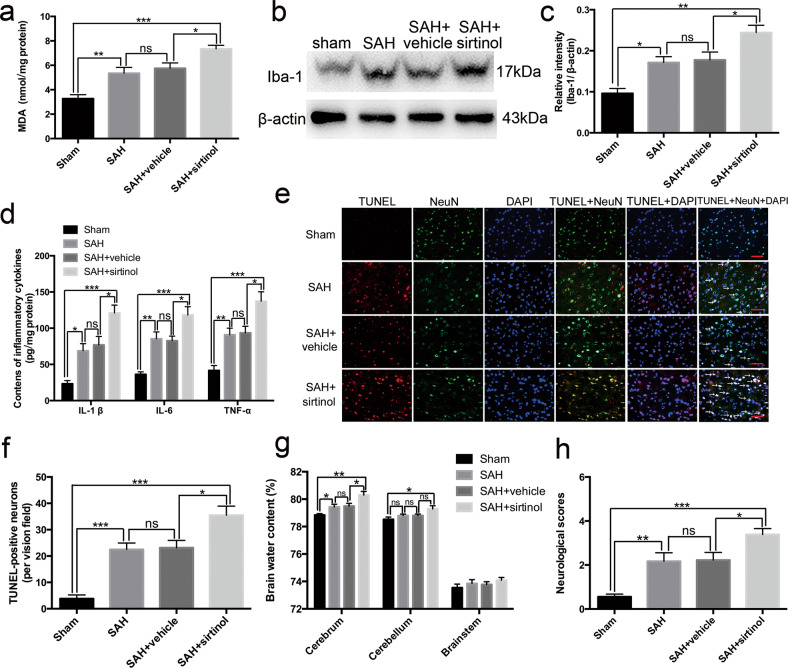# Correction: Sirtuin 1 activation protects against early brain injury after experimental subarachnoid hemorrhage in rats

**DOI:** 10.1038/s41419-022-05427-y

**Published:** 2022-11-17

**Authors:** Xiang-Sheng Zhang, Qi Wu, Ling-Yun Wu, Zhen-Nan Ye, Tian-Wei Jiang, Wei Li, Zong Zhuang, Meng-Liang Zhou, Xin Zhang, Chun-Hua Hang

**Affiliations:** 1 Department of Neurosurgery, Jinling Hospital, School of Medicine, Nanjing University, Nanjing, Jiangsu Province P.R. China; 2 Department of Neurosurgery, Jinling Hospital, School of Medicine, Southern Medical University (Guangzhou), Nanjing, Jiangsu Province P.R. China

Correction to: *Cell Death and Disease* 10.1038/cddis.2016.292, published online 13 October 2016

The original version of this article unfortunately contained an error in fig. 3e. The authors apologize for the error. The correct figure can be found below.