# Supplementary material for: Sirtuin 1 activation protects against early brain injury after experimental subarachnoid hemorrhage in rats
Source: Cell Death Dis. 2016 Oct 13;7(10):e2416–. doi: 10.1038/cddis.2016.292 (PMC5133967; doi:10.1038/cddis.2016.292)
Supplement: Supplementary Figure Legends [file cddis2016292x1.docx]

**Additional files**

**Figure 1.** Schematic illustration of experiment design. Experiment 1 (**a**) is performed to evaluate the time course of expression of SIRT1 after SAH and indicate the appropriate time point for the second experiment. Experiment 2 (**b**) and 3 (**c**) are conducted to investigate the effects of inhibition SIRT1 on EBI after SAH and indicate the potential mechanisms. Experiment 4 (**d**) is designed to evaluate whether activation of SIRT1 confers brain protection after SAH.

**Figure 2.** Schematic representation of the cortex sample area for detection. (**a**) Rat brain of the sham group and (**b**) the SAH group harvested 24 h after surgery. (**c, d**) The detected areas of the cortex sample for relative assays are illustrated.
