# Supplementary figures and images for: Sirtuin 1 activation protects against early brain injury after experimental subarachnoid hemorrhage in rats
Source: Cell Death Dis. 2016 Oct 13;7(10):e2416–. doi: 10.1038/cddis.2016.292 (PMC5133967; doi:10.1038/cddis.2016.292)

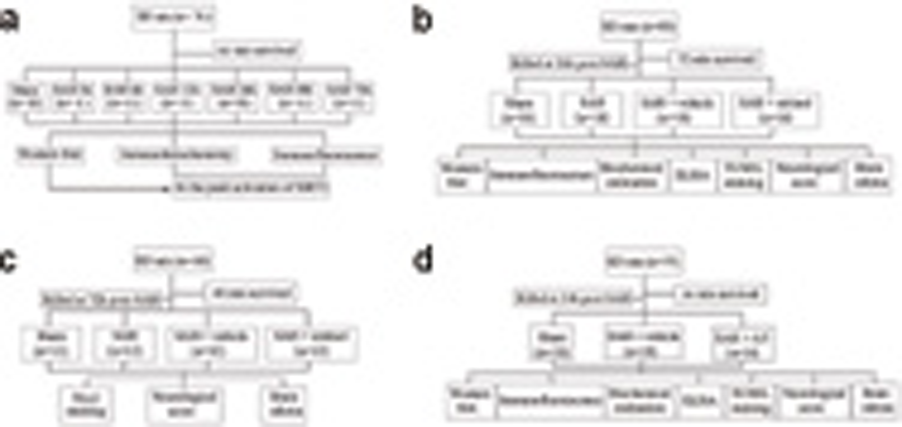

Supplement: Supplementary Figure 1 [file cddis2016292x2.tif]

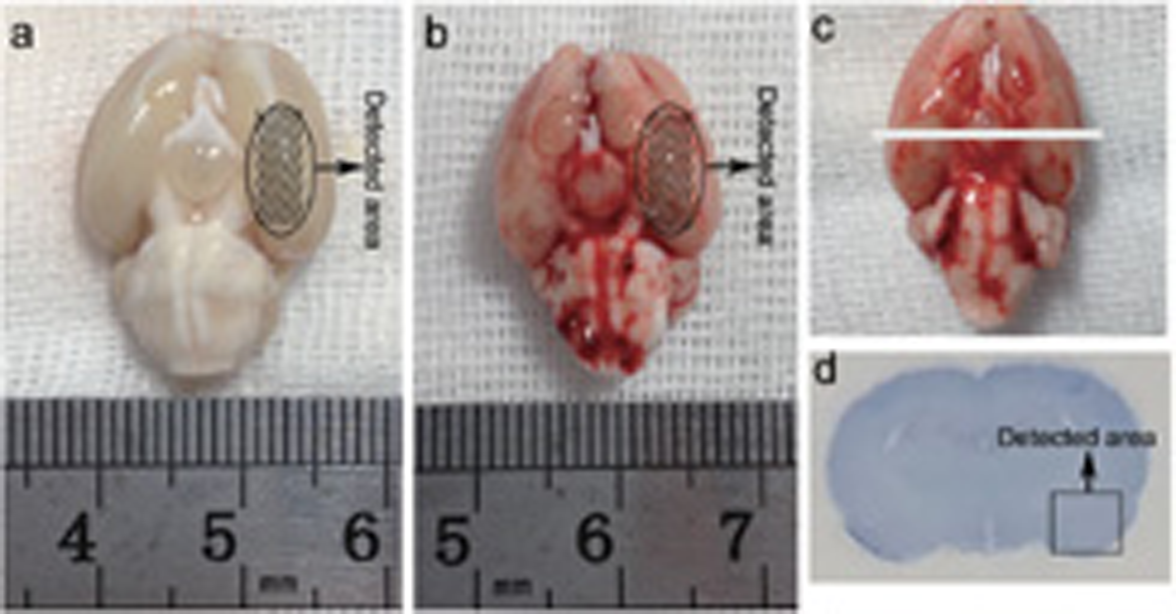

Supplement: Supplementary Figure 2 [file cddis2016292x3.tif]
